# Our State Boards

**Published:** 1901-12

**Authors:** 


### OUR STATE BOARDS.

In our November issue we published some matters of interest in connection with our State Veterinary Boards of Examiners, and in looking over the work of these boards we find that there is considerable latitude exercised by the various boards in interpreting what constitutes a legally chartered and regularly organized veterinary college. Some of the boards whose laws appear to be the most stringent and exacting place their name of approval and recognition on colleges not recognized in their own Commonwealths, and whose graduates are not eligible to membership in the American or State Veterinary Medical Associations. In Michigan we note that, while the law prescribes that all registrations of graduates are to be made within three months, the work of registration continues for two years after the passage of the law, and during which period we have no doubt that many who registered in 1901 were in daily violation of the provisions of the law during this period. We believe that there is nothing that tends to rob a law of its merits so much as to find those who are graduates of colleges, and should know better, ignoring or treating with contempt these movements toward higher veterinary education.

